# Development of the ‘Culturally Informed Ecological Model of Grief’ – a national qualitative study of bereavement experiences among ethnically diverse communities

**DOI:** 10.1186/s12916-026-05063-9

**Published:** 2026-07-14

**Authors:** Catriona R Mayland, Emily Fisher, Adejoke O Oluyase, Nikel-Shaniece Hector-Jack, Toslima Khatun, Rukia Saleem, Naureen Khan, Shirin Shahid, Riffat Mahmood, Gurpreet Grewal-Santini, Monika Afolabi, Candice Wang, Sarah Ng, Rashmi Kumar, Katherine Bristowe, Emily Harrop, Zoebia Islam, Jonathan Koffman, Gurch Randhawa, Lucy E Selman, Sabrina Bajwah

**Affiliations:** 1 School of Medicine and Population Health, University of Sheffield, Sheffield, UK; 2 Cicely Saunders Institute of Palliative Care, Policy and Rehabilitation, King’s College London, London, UK; 3 Talk, Listen, Change (TLC) Workforce Research Programme, Institute for Health and Well Being Research, University of Bedfordshire, Luton, UK; 4 St Luke’s Hospice, Sheffield, UK; 5 LOROS Centre for Excellence Community Researchers, Leicester, UK; 6 Institute for Health Research, University of Bedfordshire, Luton, UK; 7 Sheffield Chinese Community Centre, Sheffield, UK; 8 Patient and Public Involvement Representative, King’s College London, London, UK; 9 Marie Curie Research Centre, Division of Population Medicine, School of Medicine, Cardiff University, Cardiff, UK; 10 LOROS Hospice Centre for Excellence, University of Leicester, Leicester, UK; 11 Wolfson Palliative Care Research Centre, Hull York Medical School, University of Hull, Hull, UK; 12 Palliative and End of Life Care Research Group, Population Health Sciences, Bristol Medical School, University of Bristol, Bristol, UK

**Keywords:** Bereavement, Grief, Qualitative research, Ethnicity, Healthcare disparities, Racism

## Abstract

**Background:**

Among people from ethnically diverse communities, evidence regarding bereavement experiences and support needs is limited; there are known inequities in access to formal bereavement support. Our study aimed to address this gap, provide new theoretical insights into grief, and inform more equitable bereavement support services.

**Methods:**

A national, qualitative study was conducted with bereaved participants purposively sampled by ethnicity, age, sex, relationship to the decedent, and nature of death. Community Researchers from ethnically diverse communities, trained in research methods, were integral to recruitment, data collection and analysis. Focus groups and interviews were conducted in participants’ preferred language and analysed using framework analysis. A novel conceptual grief model was developed through inductive analysis.

**Results:**

One-hundred and twenty people (female = 88 (73.3%), aged 21–81 years) participated in nine focus groups (*n* = 67 participants) and 53 one-to-one interviews. Participants were of Bangladeshi, Indian, Chinese, Pakistani, Black African, Black Caribbean, Arab, White Irish or Polish ethnicity. They were bereaved due to adult or child deaths, sudden and anticipated deaths. Sixty-nine per cent were first-generation migrants, 55% had a first language other than English, and 38% did not have access to education beyond primary or secondary school.

**Conclusions:**

Racism was evident across multiple settings and closely intertwined with bereavement, compounding the emotional burden of grief. This study represents the largest qualitative investigation of bereavement among ethnically diverse communities in UK healthcare to date, providing a robust evidence base for improving equity in support. Bereavement services must prioritise cultural safety and actively address structural racism. The ‘Culturally Informed Ecological Model of Grief’ offers a new framework guiding more equitable bereavement support, relevant across national and international contexts.

**Supplementary Information:**

The online version contains supplementary material available at 10.1186/s12916-026-05063-9.

## Background

The public health toll of bereavement is a significant stressor on individuals and society [1]. Bereaved people are at a greater risk of adverse outcomes, such as anxiety, depression and prolonged grief disorder [2, 3]. They also face increased socio-economic costs, including reduced rates of employment, greater healthcare utilization [4], and increased morbidity and mortality, especially in the first year after the death [5, 6].

Crucially, emerging evidence suggests that structural inequalities, particularly those related to race and ethnicity, shape these impacts [7]. In the United States of America (USA), Black Americans experience the loss of family and friends more frequently than white Americans due to shorter life expectancy [7]. During the COVID-19 pandemic, ethnically diverse communities in the United Kingdom (UK) experienced disproportionately high death rates, often enduring multiple losses [8]. In addition, inequities exist in access to bereavement support services for people from ethnically diverse groups. A previous UK qualitative study suggested ethnically diverse groups may experience higher levels of shame and stigma following certain types of death, such as suicide, while lacking access to culturally-specific support [9]. Furthermore, in a survey of UK voluntary and community sector bereavement services (*n* = 147), over a third of services reported they did not reach people from ethnically diverse communities who needed additional support [10]. Qualitative survey findings demonstrated the disproportionate impact of the pandemic on minoritised ethnic communities, disrupting care and mourning practices. Yet while participants recognised the need for culturally appropriate support, outreach to ethnically diverse communities was poor [10].

A systematic review identified additional barriers to accessing support, including the medicalisation of grief, inadequate tailoring of services to meet the needs of ethnically diverse communities, and limited cultural competency training among service providers [11]. The review also highlighted a lack of evidence to inform service provision, particularly about ethnically diverse communities’ bereavement experiences and the care provided by family, friends, social and faith-based support systems [11].

Existing psychological models of grief [12–14] have generally been developed in countries such as the USA and the UK, reflecting viewpoints shaped by specific cultural lenses that incorporate historical experiences and values e.g. individualism. These models often fail to account for the diversity of grief that different cultural groups experience. For example, the ‘Dual Process Model’ [13] describes grief as an oscillating process in which individuals move between loss-orientated coping (thinking about the death and the emotional pain) and restoration-orientated coping (focused on rebuilding life and adapting to new circumstances). Similarly, ‘Meaning Reconstruction’ [15] emphasises how individuals create new meaning to make sense of the loss. Neither of these models, however, fully capture the racialised, relational and community-driven aspects of grief.

Although researchers did not originally develop it in relation to bereavement, Bronfenbrenner’s Ecological Systems Theory offers an approach that addresses some of these limitations [16]. This theory explains how a network of interconnected environmental factors shapes individual development, ranging from immediate surroundings such as family to larger societal structures like culture [16].

The theory conceptualises these factors as five interrelated systems: the microsystem (immediate relationships and settings), mesosystem (connections between these settings), exosystem (indirect environmental influences), macrosystem (broader cultural and societal values), and chronosystem (changes over time). Together, these systems provide a framework for understanding how multiple contextual factors shape individual experiences [16]. Researchers have previously used the theory to conceptualise children’s experiences of grief during the COVID-19 pandemic, the complexity of grief at work, and children’s development within the context of migration [17–19]. Hence, it is well placed to conceptualise the many different components of identity, affiliation and culture influencing bereavement experiences.

In view of the lack of research available to inform service provision for ethnically diverse communities, this study is part of a wider research project to address evidence gaps and inform the development of more equitable, effective bereavement support services [20]. We aimed to explore experiences of bereavement amongst ethnically diverse communities in England and, by applying the ecological framework to our findings, generate a new theoretical model to reflect these experiences.

## Methods

We report the study in accordance with the COnsolidated criteria for REporting Qualitative research (COREQ) guidelines [21].

### Defining key terms

Within the context of this study, the term ‘ethnically diverse communities’ includes all who self-identify as being from an ethnic group other than ‘white British’. We agreed upon this as an acceptable, non-stigmatising term, (compared with ‘ethnic minorities’), through collaborative discussion with our research advisory team. The term ‘ethnically diverse communities’ relates to groups of individuals connected through shared ethnic, cultural, linguistic, religious, and/or migration-related identities, rather than solely geographical location. This term allows for flexibility and self-interpretation. In terms of defining different ethnicities, the UK Census categories were used and all groups were included except for ‘white British.’ [22].

### Study design and setting

We conducted a cross-sectional, qualitative study involving one-to-one semi-structured interviews and focus groups with bereaved people from ethnically diverse communities in England. The four main study sites were London, Sheffield, Leicester and Luton, chosen due to their geographical breadth and variety of ethnically diverse communities. Data collection occurred between January and October 2024. The ethnically diverse, multidisciplinary research team had expertise in bereavement and ethnicity research, working with vulnerable populations, Patient and Public Involvement (PPI) representatives and qualitative research. We adopted a social constructivist theoretical approach, that recognises knowledge, reality and understanding are not inherent but rather actively constructed through social interaction and cultural context, including historical and political processes [23]. We consulted our research advisory group, which met every 4–6 months throughout the project, for key decisions, and included PPI representatives, representatives from different charities, faiths, bereavement support organisations and local councils.

### Eligibility criteria

Inclusion criteria were adults (≥ 18 years old) who self-identified as being from an ethnically diverse community, had experienced a bereavement (any cause of death; any relationship to the deceased), resided in England and were able to provide informed consent (in any language). We excluded potential participants if they had experienced a bereavement within the last eight weeks (unless they expressed interest in the study before this time). As bereavement is a salient event [24], we placed no other restrictions on the time elapsed between experiencing the death and participation.

### Sampling

We used a purposeful sampling strategy, initially identifying and selecting potential participants who met predetermined criteria e.g., specific ethnically diverse groups. Within the four main study sites, potential participants were first identified from the following communities (reflecting groups from the UK Census data): Black African, Black Caribbean, Chinese, Indian, Bangladeshi, Pakistani, and Polish. We then used maximum variation sampling [25], aiming to capture diversity in: ethnicity, age, sex, religion, and the nature of the death. This approach considered key dimensions of variation and helped determine which groups were represented and where ‘gaps’ existed that could be targeted.

### Recruitment

Our approach used the ‘Respect, Trust and Equity’ model of recruitment, to be published separately (Bajwah S, personal communication) [26]. This model embeds ‘cultural safety’ [27] and an asset-based recruitment strategy through the key principles of respecting participants, acknowledging historical injustices and actively addressing power imbalances. We linked with local community groups via eight paid bilingual ‘Community Researchers’ - people from specific ethnically diverse communities - who received bespoke research methodology training. They were supported to conduct interviews and/or co-facilitate focus groups and were fully integrated into the research team. The training programme included introductory sessions on research principles, alongside practical skill-building in conducting interviews and facilitating focus groups. It also covered research ethics, as well as approaches to managing sensitive conversations and participant distress.

Key contacts, such as heads of community organisations and religious leaders, passed on recruitment materials and, sought verbal consent for the study team to contact interested potential participants. We also recruited participants via the ‘**KE**ech **E**nd of life research **P**artnership **NET**work’ (KEEPNET) [28], which aims to build research capacity and capability with ethnically diverse communities. Additional recruitment methods included word-of-mouth, social media, emails, posters, and through personal and professional networks. Potential participants were informed that the study’s aim was to understand bereavement experiences among ethnically diverse communities and improve support services.

Participant Information Sheets, containing both visual and written information, were available in English, Cantonese, Bengali, Urdu and Somali, both electronically and in paper form. Telephone and email contact details for the research team were provided and individuals were invited to express interest directly, via a Community Researcher or a gatekeeper.

### Consent

Depending on participant preference, consent was given using online or written consent forms or recorded verbally; where needed, interpretation was provided by Community Researchers.

### Data collection

Interviews took place face-to-face (at a location chosen by the participant) or via video call. Although the option of telephone interview was available, no participant requested this. Focus groups were face-to-face (at local community centres) or online. We discussed and facilitated participant choices about the means of data collection where possible, balancing this with practical factors such as geographical location. Interviews and focus groups were conducted in languages other than English as needed. We developed and piloted the topic guides (Additional file 1) with PPI representatives and key stakeholders, informed by the research aims and relevant literature. Questions focused on personal bereavement experiences, including informal and formal support; views about appropriateness, accessibility and acceptability of bereavement support services; and suggestions to improve support. Focus groups and interviews were facilitated by experienced qualitative researchers (CRM, SB, AOO, EF, ZI) and/or at least one of the Community Researchers (NK, CW, SN, RS, SS, RM, GG-S, MA), who interpreted as needed. For some people, this was the first time they had spoken in a group setting about grief and loss. Therefore, discussions had to be carefully supported by trusted individuals, and a flexible approach was needed in terms of time and location. Researchers made field notes immediately afterwards. Participants were asked for demographic details (self-identified ethnicity, age, sex, religion) to contextualise our sample.

### Analysis

Interviews and focus groups were digitally recorded, transcribed *verbatim*, checked for accuracy and, where needed, professionally translated into English and re-checked by local site leads and community researchers prior to analysis. All transcripts were anonymised. NVivo (version 12) was used to facilitate data management. We used a five-stage Framework Analysis approach [29]. Firstly (i), members of the research team (EF, CRM, SB, LES, JK, AOO) familiarised themselves with the data, including both transcripts and field notes. Then (ii), a thematic framework was created by team members independently coding a sample of initial transcripts. We used a combination of inductive and deductive coding, informed by the study objectives. The team met to discuss immediate impressions and initial coding. This informed the creation of a draft coding framework focusing on bereavement experiences and facilitators, barriers and potential solutions to providing bereavement support for ethnically diverse communities. The draft coding framework was then applied (iii) to selected transcripts (EF) and further refined. Research team members (EF, CRM, SB, LES, JK, EH, AOO, KB), with input from Community Researchers (RS, GG-S), reviewed and finalised the coding frame which was then applied to all transcripts (CRM, EF, AOO, NH-J, TK). We summarised and charted data (iv) (CRM, SB, AOO, NH-J, TK) to both reduce the data, whilst retaining the original meaning, and highlight illustrative quotations. Findings were discussed and refined with the wider team and the Community Researchers (RS, GG-S, NK). Finally (v), charting was used to map out phenomena of interest, in keeping with the study aims and objectives, and identify relationships between themes to explicate findings. Unique ID codes are used in reporting, denoting data type (P = interview participant; FG = focus group number) and site number. Quotations were in English unless stated otherwise. Within the participant labels for exemplar quotations, where a participant was the sole person from their ethnically diverse community, to protect anonymity, their ethnicity is not reported.

Following the framework approach to analysis, the Bronfenbrenner’s Ecological Systems Theory [16] was used as a lens to map and describe the individual, relational, environmental and systemic components which influence grief experiences. The team engaged in cross-case comparison and analytic mapping to identify relationships between individual, social, and structural experiences of grief. We collaboratively explored patterns that cut across ecological levels to refine interpretive categories and support the development of the conceptual framework.

### Positionality

The research team comprised academic and community researchers with diverse cultural and ethnic backgrounds. Reflexive discussions about language, power dynamics and racism, occurred throughout data collection and analysis shaping interpretation, particularly around how insider and outsider perspectives influenced meaning-making. Multilingual analysis sessions captured cultural nuances in grief language and religious interpretations, contributing directly to the model’s conceptual development. A Community Researcher who was also a Muslim scholar, co-facilitated three focus groups comprised of individuals of Muslim faith. This enabled them to lead discussions within the context of Quranic teachings, which helped group members relate to the concept of bereavement, especially if there wasn’t a directly translatable term.

This reflexive approach aligns with our constructivist epistemology, viewing knowledge as co-produced and contextually situated rather than objective or neutral. Additionally, it aligns with recently established consensus-based principles for developing, undertaking, and reporting research with minority ethnic populations in palliative and end of life care, which emphasise trust-building, power-sharing, reflexivity, and transparent reporting [30].

## Results

### Participant characteristics

One hundred and twenty-one participants took part in nine focus groups (*n* = 67 participants) and 54 individual interviews (female = 89 (73.6%), age range 21–81 years) (Table 1, Additional file 2). Participants were bereaved of both children and adults, with deaths due to diseases or chronic illnesses, as well as sudden deaths from trauma or by suicide, represented. Participants came from a wide range of ethnically diverse communities including, Bangladeshi, Indian, Chinese, Pakistani, Black African, Black Caribbean, Arab, White Irish, and Polish. Sixty-nine per cent were first-generation migrants, 53% had a first language other than English, and 38% did not have access to education beyond primary or secondary school. Muslim was the most frequently reported faith (*n* = 59, 48.8%). Interviews lasted approximately 60 min (range 28 to 136 min) and focus groups ranged from 53 to 104 min.

**Table 1:** Demographic characteristics of participants

Characteristics (*n* = 121)	*n*	%
*Participant*		
Sex Female Male	89 32	73.6 26.4
Age 21–40 41–60 61–80 81 or over	36 61 23 1	29.8 50.4 19.0 0.8
Ethnicity Bangladeshi Black African Pakistani Chinese Indian Black Caribbean Any other Asian background Any other Black, Black British, or Caribbean background Any other White background Any other Mixed or multiple ethnic background Arab White - Irish White and Asian Missing	28 19 20 16 12 8 7 3 3 1 1 1 1 1	23.1 15.7 16.5 13.2 9.9 6.6 5.8 2.5 2.5 0.8 0.8 0.8 0.8 0.8
Place of birth Outside the UK UK Missing	83 35 3	68.6 28.9 2.5
First language English Other Missing	55 64 2	45.5 52.9 1.70
Language if other (reported as % of whole sample) Bangla Cantonese Polish Gujarati Punjabi Yoruba Arabic Chinese Guajarati Hindi Mandarin Somali Urdu Urdu/Punjabi Missing	20 20 3 2 2 2 1 1 1 1 1 1 1 1 7	16.5 16.5 2.5 1.7 1.7 1.7 0.8 0.8 0.8 0.8 0.8 0.8 0.8 0.8 5.8
Faith/religion Muslim Christian No religion Hindu Sikh Faith/religion (continued) Buddhist	59 35 13 9 4 1	48.8 28.9 10.7 7.4 3.3 0.8
Highest education level Primary school High School/Secondary School completed/diploma Bachelors/master’s level Doctoral Level Missing	13 33 66 7 2	10.7 27.3 54.5 5.8 1.7
Occupation Modern professional & traditional professional occupations Senior, middle or junior managers or administrators Doctoral Level Clerical and intermediate occupations Technical and craft occupations Routine, semi-routine manual and service occupations Small business owners Long-term unemployed Retired Full-time carer Other Missing	34 13 1 8 2 6 6 14 21 9 1 6	28.1 10.7 0.8 6.6 1.7 5.0 5.0 11.6 17.4 7.4 0.8 5.0
*Person who has died*		
Sex Female Male	50 70 1	41.3 57.9 0.8
Age Up to 20 21–40 41–60 61–80 81 or over	4 11 35 51 20	3.3 9.1 28.9 42.1 16.5
Ethnicity Bangladeshi Black African Pakistani Chinese Indian Black Caribbean Any other Asian background Any other White background Any other Black, Black British, or Caribbean background Any other Mixed or multiple ethnic background White - English, Welsh, Scottish, Northern Irish or British White - Irish Arab Black, Black British, Caribbean, or African Missing	28 20 19 16 11 8 5 3 2 2 2 2 1 1 1	23.1 16.5 15.7 13.2 9.1 6.6 4.1 2.5 1.7 1.7 1.7 1.7 0.8 0.8 0.8
Place of birth Outside the UK UK Missing	100 20 1	82.6 16.5 0.8
First language English Other Missing	41 74 6	33.9 61.2 5.0
First language if other (reported as % of whole sample) Bangla Cantonese Gujarati Punjabi Urdu Polish Yoruba Arabic Hindi Japanese Patois Somali Urdu/Potwari Urdu/Punjabi Missing	25 19 4 4 4 3 2 1 1 1 1 1 1 1 6	20.7 15.7 3.3 3.3 3.3 2.5 1.7 0.8 0.8 0.8 0.8 0.8 0.8 0.8 5.0
Faith/religion Muslim Christian No religion Hindu Buddhist Sikh Other religion	60 31 14 8 4 3 1	49.6 25.6 11.6 6.6 3.3 2.5 0.8
Education No education Primary school High School/Secondary School completed/diploma Bachelors/master’s level Doctoral Level Missing	1 30 44 31 3 12	0.8 24.8 36.4 25.6 2.5 9.9
Occupation Modern professional & traditional professional occupations Senior, middle or junior managers or administrators Doctoral Level Clerical and intermediate occupations Technical and craft occupations Routine, semi-routine manual and service occupations Small business owners Long-term unemployed Retired Full-time carer Other Missing	23 6 0 3 7 13 7 9 30 7 2 14	19.0 5.0 0.0 2.5 5.8 10.7 5.8 7.4 24.8 5.8 1.7 11.6
Occupation if other Housewife Student	1 1	0.8 0.8
Social circumstances at time of death Living with family Living alone at home Residential/Nursing Home Other e.g., working overseas Missing	99 10 6 4 2	81.8 8.3 5.0 3.3 1.7

### Findings

**Fig. 1:**
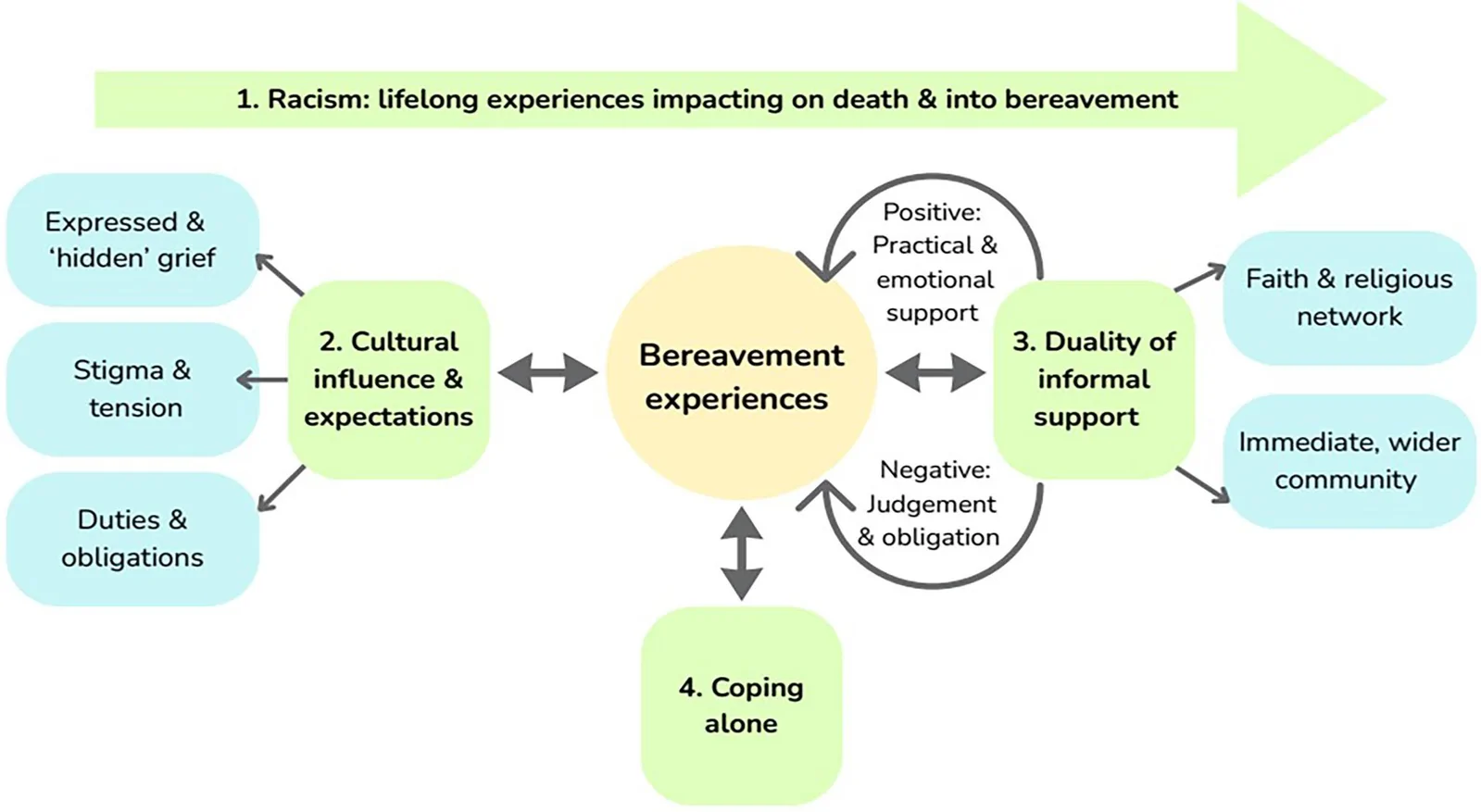
Bereavement experiences

We describe four main themes related to participants’ bereavement experiences, with sub-themes as follows: (1) Racism, (2) Cultural influence and expectations (Expressed and hidden grief, Stigma and tension, Duty and obligations), (3) Duality of informal support (Faith and religious network, Wider community), and (4) Coping alone Fig. 1.

#### Racism

Participants from a wide range of ethnically diverse communities (including Polish, Black Caribbean, Indian, Chinese and Arab) described experiences of differential or exclusionary treatment linked to their ethnicity, nationality, language, or cultural identity. These experiences occurred throughout their lives, including during end-of-life care and into bereavement. In some cases, participants explicitly identified these experiences as racism. In other cases, we interpreted these experiences within the analytic framework as racism, based on participants’ descriptions of ethnicity-related discrimination. Participants shared lifelong exposure to interpersonal racism (direct encounters occurring at an individual level) across multiple settings including social care, healthcare and employment, which continued into their bereavement and created an additional layer of trauma. Some, like this participant, described overt racist remarks relating to their presumed ethnic origin, which undermined their rights to support.

*‘The man at the job centre said that if I could not speak English*,* I should go back to Poland. I felt terrible.’* (Site 4, P21, female, interview conducted in Polish).

Other people experienced more subtle forms of discrimination that, although less explicit, were equally harmful. One example, discussed within a focus group of predominantly Chinese participants, related to managers at work lacking appreciation of the cultural significance of funeral attendance. Participants were left wondering if there was an ‘*element of racism*’ as they perceived a ‘*British colleague’* would not be treated in the same manner. They reflected on discrimination as follows:

*‘Unfortunately*,* these days*,* racial discrimination is very subtle. And so*,* if (participant’s name) feels uncomfortable*,* then that is enough… Nowadays*,* racism has become very*,* very subtle. It’s not blatantly obvious to the face like 40*,* 50 years ago.’* (Site 1, FG2, male & female participants, Chinese & ‘Any other Asian background’).

Implicit racism exacerbated feelings of discomfort and alienation that made participants question their own experiences, with some unsure whether their treatment was racially motivated or coincidental.

Participants described examples where their healthcare treatment – or the treatment of their family around the time of death – was affected by institutional racism (ingrained biases within institutions contributing to unfair treatment). This included a Black Caribbean woman’s account that their family member (and ‘*another black patient’*) were neglected by nursing staff, due to their ethnicity, after sustaining a fall.

*‘During the time that she (participant’s sister) was in hospital*,* she experienced so much racism from the nurses who were white and Filipino and Asian. It was clear that they had a problem with black patients on this ward.’* (Site 1, P05, female, Black Caribbean).

Another participant recollected the incorrect assumptions that healthcare professionals made about their Hindu faith. These experiences eroded trust in the system and compounded the emotional trauma of an already distressing situation.

*‘So*,* it was probably about six hours before my dad died*,* the chaplain sent across someone who looks like us because they’re brown. But then we realised that he was an imam……for them to not know the difference even was pretty upsetting and frankly*,* a little bit insulting.’* (Site 1, P04, female, Indian).

Assumptions, conscious and unconscious bias related to cultural or personal identity further marginalised participants. These experiences influenced how participants engaged with support services, leaving many feeling unwelcome or unsafe in formal support spaces.

*‘I wanted to be part of the community because I wanted to find*,* like*,* a safe haven*,* like*,* to surround myself. Sometimes I wasn’t… accepted because people still have stereotypes… they won’t accept you for what you are or what you look [like].’* (Site 2, P14, female).

Furthermore, experiences of racism led to disillusionment with organisations, with some participants feeling that institutional racism was too deeply entrenched to change.

**‘***You have to dismantle the racism before you can find a way to make these sorts of services work… That’s not going to happen anytime soon.’* (Site 1, P05, female, Black Caribbean).

Even in spaces designed for emotional support, participants felt unable to openly address the racism they had previously experienced. They explained that therapists avoided or dismissed discussions about race, citing situations where discussions were curtailed or not followed up, which participants linked to organisational constraints or practitioners’ concerns regarding how to respond.

*‘I recorded him (counsellor) saying to me*,* ‘You can talk about your sister dying*,* but you won’t be able to talk about the racism.’* (Site 1, P05, female, Black Caribbean).

It was evident that once organisations providing bereavement support lost the trust of their communities, this impacted on individuals’ willingness to engage with services.

#### Cultural influence and expectations

The second theme describes shared beliefs about means of communication, roles and behaviours that are learned from family and society. These can both support a sense of belonging but can also potentially limit individual expression. There were three subthemes within this central theme: (a) Expressed and hidden grief, (b) Stigma and tension, and (c) Duty and obligations.

##### Expressed and hidden grief

All participants expressed a range of emotions relating to their grief, including sadness, disbelief, blame, guilt, anger, and denial. One participant shared their anger following the bereavement:

*‘I was ranting*,* mentally ranting at God. Saying: you had no business. You had no business doing this. And I know*,* I know*,* I was thinking how is this fair. I’m the older sister. I should have gone first.’* (Site 2, P10, female, Indian).

Another participant shared their distress and disbelief following the death of their child:

*‘So*,* I literally spent two weeks wailing and crying and waking up in the middle of the night crying*,* because I knew that he wasn’t there anymore. And all I kept saying was*,* well*,* he’s my boy*,* he’s my boy. I couldn’t believe that he was dead.’* (Site 4, P10, female, Bangladeshi).

Participants used different analogies to share the traumatic personal impact of the death and how this had affected them, for example, having their *‘whole world turned upside down’*, ‘*losing a limb*’ or *‘ripping off a part of me’*. One participant described the collective loss to the community that the death symbolised, i.e., a loss of cultural knowledge as members from older generations died.

*‘We are lost. We’re lost about where we take ourselves and our community now. Our grief is not just immediate. There is a bigger grief going on here. There is a loss of culture. There is a loss of knowledge. There is a loss of the community who would ordinarily support us through our grief.’* (Site 2, P17, female).

An individual’s culture could affect the extent to which they were able to express their grief. Some participants shared the view that there was almost an unwritten rule that grief should be hidden and not openly discussed; there was an ‘*unspoken something*.’ These views were expressed most frequently by participants from Pakistani, Muslim communities, but also by some participants from Black Caribbean, Irish and Chinese communities, as illustrated by the following examples:

*‘I feel like*,* especially in Pakistani Muslim families*,* this concept of grief just doesn’t exist. It just gets buried.’* (Site 1, P01, male, Pakistani).

*‘It’s very difficult for Chinese or maybe Hong Kong people to talk about death…’* (Site 1, FG2, male & female participants, Chinese & ‘Any other Asian background’).

##### Stigma and tension

Whereas ‘hidden grief’ focuses on cultural limitations placed on the expression of emotions following a death, there was also specific stigma relating to communication about mental health issues and associated with specific types of death. Black African, Black Caribbean, Bangladeshi, Indian and Pakistani participants reported that mental health problems were often denounced in their communities. This then affected how openly individuals discussed their feelings, as illustrated by the following two participants.

*We don’t openly say that I’m depressed. We don’t openly say that I’m mentally ill. We don’t openly say that because of the death in the family*,* that person is vulnerable. Our culture is still hiding.’* (Site 2, P05, female, Indian).

*‘…with that word*,* ‘mental health’*,* comes this backdrop of a stigma of probably you have psychological problem*,* you know…there still needs to be reorientation that it’s*,* it’s not all bad……’* (Site 1, FG1, male and female participants, Black African).

For certain types of death, for example death by suicide, the shame and stigma, specifically mentioned in relation to Islam, meant individuals could feel isolated and unsupported. Participants shared their experiences and expressed a need for communities to address these stigmas so that support needs could be met.

*‘I actually started researching and looking at how many other Muslims have been through this*,* have experienced or are survivors of suicide bereavement. And I could hardly find any. Because I think a lot of people don’t talk about it.’* (Site 4, P24, female).

Participants also reported gender-based stigmas. Men reported the expectation to be stoic and not express their grief or emotion.

*‘…he had the notion that you’re a man*,* you shouldn’t cry. It’s just you’ll get over it.’* (Site 1, FG4, male & female participants, Black Caribbean & ‘Any other Mixed or multiple ethnic background’).

There was recognition, however, that there may be support for Muslim men within the mosque, but this was less accessible for women. There were other social and cultural taboos for women. For example, one female Sikh participant shared her experiences, as well as those of her friends, of receiving judgement for being perceived to have ‘*moved on’* too quickly after the death of their husband. Her desire to continue with life and have children was not supported.

*‘The judgment came pretty quick*,* which was my story quite a lot. And I know a lot of young widows have faced the same. Wanting to move on. I mean*,* in all aspects*,* I wanted to move on. I wanted a child. I wanted these things I never had.’* (Site 1, P14, female, Indian).

##### Duty and obligation

Cultural expectations placed upon people by their families or wider society also influenced grief. Participants reflected on the responsibilities they assumed following the death, attributing these to alterations in the family structure and relational dynamics. Certain cultural expectations, expressed for example, by Black African, Black Caribbean, and Bangladeshi participants, could be placed on family members. For example, the eldest child was ‘*expected to take the lead’* in the event of a parent’s death. Participants perceived caring for their parents as a mark of respect, a means of returning thanks for all their parents had done, and hence, a responsibility that participants welcomed.

*‘I never felt it as a burden….I didn’t feel bad….he was my father and it was my responsibility.’* (Site 3, FG1, male participants, Bangladeshi; conducted in Bengali).

These responsibilities could include provision of emotional support for the wider family, being ‘*physically present*’, and taking a lead role in sorting the practical and financial arrangements directly related to the death and in the period afterwards. Participants referred to being the ‘*backbone*’ or ‘*pillar of the family.’* At times, however, these responsibilities could be overwhelming, in terms of the level of accountability expected and the pressure this could bring. On occasion, this could mean participants didn’t have time or space to process and explore their own grief as this focus group with Black Caribbean participants discussed:

*‘…culturally within African Caribbean families*,* a lot is expected of the eldest child to take the lead. And that’s what I found quite pressurising. But you just got on with it. There was no questioning or anything. And there was nobody to say*,* well*,* I’m actually finding this quite overwhelming.’* (Site 1, FG4, male & female participants, Black Caribbean).

#### Duality of informal support

The third theme shaping bereavement experiences is related to informal support and the contrast between the benefits and the hindrances that could be placed on participants. Initial help often came from the immediate family and was generally regarded as beneficial and supportive. Other means of informal support included those provided by friends and work colleagues, creative outlets e.g., poetry groups, and online resources. Two subthemes were identified, reflecting the duality of support received by participants’: (a) Faith and religious network, and (b) Wider community, and whilst both means could be supportive, they also could be unhelpful.

##### Faith and religious network

Participants practising Islam, Christianity, Sikhism and Hinduism reported the benefit which came from their faith, as illustrated by these two participants.

*‘So*,* I found resilience in my faith. Had it not been for my faith*,* I don’t know where I would have been today*,* because there are moments where you feel like what is the point of me living.’* (Site 4, P04, female, Bangladeshi).

*‘They came and did the prayers of the sick. And they came afterwards to help. You know*,* the priest was very helpful afterwards with making the arrangements. He took a lot of time to explain everything. And that was a source of support.’* (Site 2, P17, female).

Even in situations where communities subscribed to the same religion, religious practices were adapted in accordance with distinct cultural norms and traditions. The positive means of support were especially reported by those of Muslim faith. Prior to the death, the ‘*Islamic signs’* informed participants about changes to expect when someone was dying, which was deemed helpful in terms of preparedness. After the death, participants reported that faith-based instructions, such as regular prayer, was an ‘*anchor*,’ helping provide structure during bereavement and a connection with the deceased. Having an understanding or appreciation of an afterlife and a higher power was a source of comfort and helped provide meaning when death occurred. Some participants, however, reported that intense feelings of grief led them to question their faith, or that their faith had been weakened because of their bereavement.

*‘But when I lost my son*,* I did not just lose my son. I lost my faith. I lost my belief. I didn’t go to Gurdwaras for donkeys*,* for decades after that*,* to be honest with you.’* (Site 3, FG2, female, Indian, Pakistani & Bangladeshi participants; conducted in English & Bengali).

For a minority of Muslim participants, religion brought judgement, with the death taken as divine retribution on those whose religious actions were deemed insufficient.

*‘And you know*,* I sort of turned to them and*,* you know*,* they’ve been my friends since I was really*,* really young. And I remember one of them saying to me*,* oh*,* do you know*,* this is all happening to you because you don’t practice your religion enough.’* (Site 4, P14, female, Pakistani).

Additionally, some participants perceived that the support offered by their religious organisation was limited or their leaders were ill-equipped to understand their specific needs.

*‘We can’t go to the mosque and talk to the Islamic leaders about losing our loved ones*,* or they may not be qualified in this area—they might not know how to provide support in these situations. They aren’t trained to offer mental support to someone who’s grieving…’* (Site 2, FG1, female, Bangladeshi).

##### Wider community

Participants reported both positive and negative aspects of support from their wider communities. Positive aspects included emotional support through conversations about the deceased and practical tasks such as shopping, bringing food, taking on chores, helping with funeral plans or providing advice, as this Somali focus group shared:

*‘If someone close to us dies*,* when it happens to us*,* we support each other. We bury the person together. People come together. Food is brought for those who come from far places.’* (Site 2, FG3, male & female, Somali; conducted in Somali).

Another specific example was given where the Pakistani community had taken on collective responsibility related to raising funds to enable a family to repatriate their relative’s body.

For a minority of participants, from mixed Asian, Pakistani and Black African communities, however, the tradition of the wider community showing their respects could be overwhelming. Participants perceived that obligation drove this tradition rather than the desire to provide meaningful support to those bereaved.

*‘….they’re not there to listen to you cry or support you. They’re just there because of this obligation. Oh*,* this person’s died. Now we have to be seen to go and visit…’ (Site 2*,* P01*,* female*,* ‘White and Asian’)*.

Additionally, it didn’t allow participants time or space alone to help process and manage their grief.

*‘Everyone turned up*,* their daughter-in-laws*,* nieces*,* like why? And it had only been about an hour. There was no need for them to come and I went in there…… I didn’t want to see them and then when I went there*,* that made me more angry*,* and I just had to bottle my feelings. I couldn’t cry*,* I just sat there but then all I could feel was people staring*,* like looking at me*,* thinking*,* oh my God*,* why is she not crying?’* (Site 4, P14, female, Pakistani).

Furthermore, participants provided examples of comments made by the wider community that could be insensitive or associated with judgement.

*‘…I was shocked that people who know religion and they know things*,* they still use such words*,* or they still ask such questions. How old are you? How old was he? What’s the age gap? Why was there so much age gap?…….we had our marriage was all for almost 10 years only. Oh*,* so. Oh*,* you must not be that sad because it’s only 10 years. Like*,* oh*,* my God. Some of the things that people said that they commented were abhorrent. They were actually abhorrent.’* (Site 4, P15, female, Pakistani).

#### Coping alone

Across several ethnic groups, including the Chinese, Polish and Arab communities, some participants described unique experiences of coping with grief in isolation, often shaped by migration, language barriers, cultural unfamiliarity, and limited access to culturally sensitive support.

*‘If we can handle it ourselves*,* it’s fine. We just don’t want to trouble people. Because when you deal with these things*,* you have to ask others for help*,* especially when we don’t know the local culture or language’* (Site 2, P04, female, Chinese; conducted in Chinese).

The accounts revealed both self-reliance and disconnection, a determination to manage grief privately, but also an underlying sense of loneliness and marginalisation. For many, coping alone reflected necessity rather than choice. Participants shared their sense of being an outsider, especially if they had recently moved to England, being isolated and feeling they had no option but to cope alone.

*‘For the first time*,* someone close to me died in this country where I am a stranger. I did not know where or what*,* so I said no*,* thank you and had to cope on my own’* (Site 4, P21, female, ‘Any other white background’; interview conducted in Polish).

A further participant, who experienced a bereavement shortly after moving to England, reported having a limited support network, being conscious that they weren’t part of the dominant culture and felt adrift from their usual mechanisms of support.

*‘I wanted to feel accepted… to find people who look like me*,* who speak my language.’* (Site 2, P14, female).

This led the participant to setting up a support network for people of their community who might subsequently find themselves to be in a similar situation.

Many participants sought solace in communities that shared their cultural, linguistic, or racial identity and actively looked for this with variable results. Comparisons were made with the support they would expect ‘*back home*,*’* which was often community-driven. Some communities had no prior awareness of the concept of formal support services. Even in situations where participants wanted to access formal bereavement support, such services were widely regarded as insufficiently visible.

*‘Do they exist*,* the bereavement services? Then there’s your answer. Nobody knows about it.’* (Site 4, P19, female, Pakistani).

### Development of the ‘Culturally Informed Ecological Model of Bereavement’

**Fig. 2:**
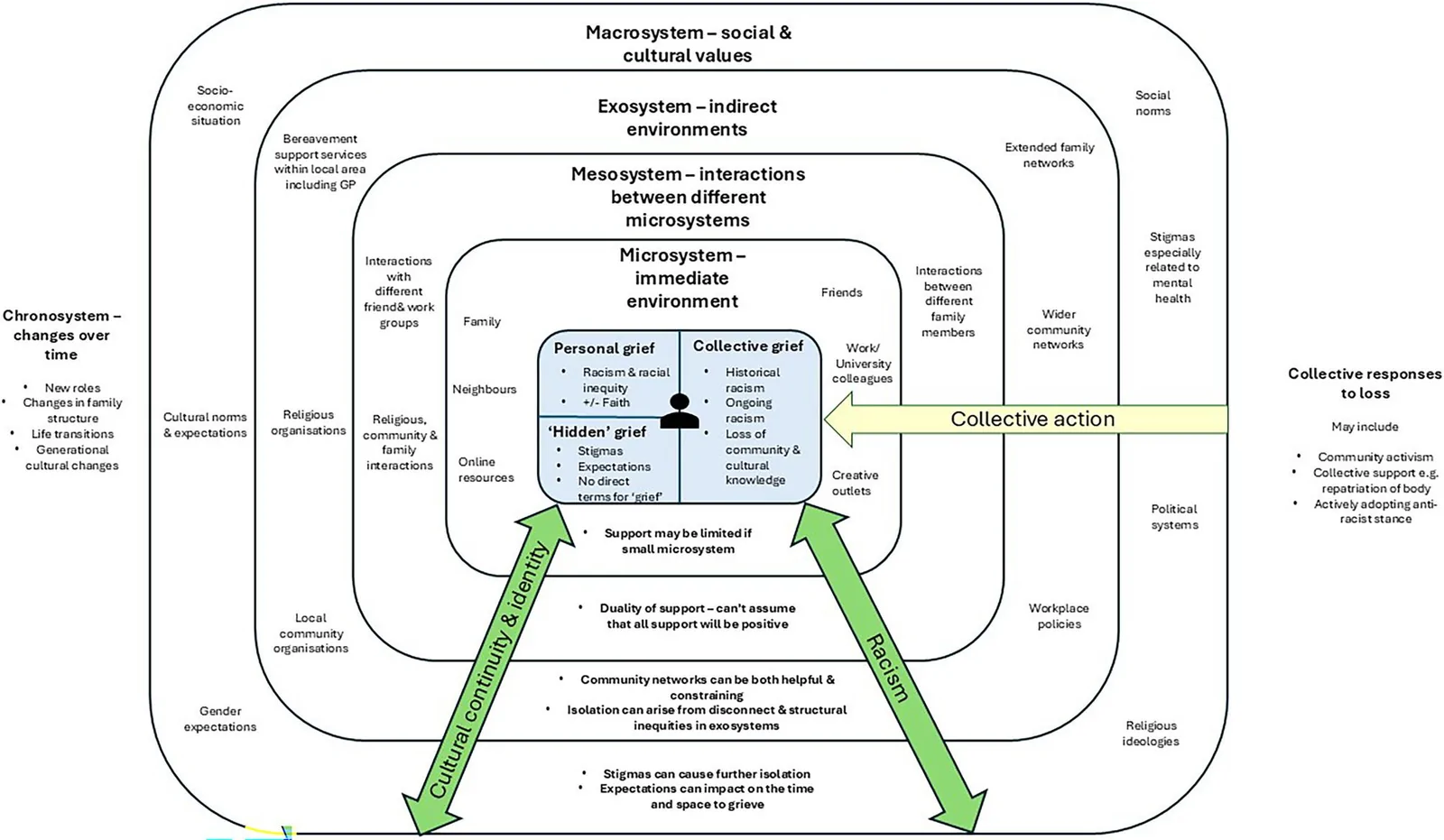
Culturally informed ecological model of grief

The thematically derived data were mapped, using an ecological framework, to develop the ‘Culturally Informed Ecological Model of Grief’ (Fig. 2). This new conceptual model frames grief as a racialised socio-relational experience operating across personal, community, institutional, and societal systems.

## Discussion

### Summary of main findings

In this large, in-depth qualitative study of bereavement experiences among ethnically diverse communities in England, we identified a complex interplay of cultural, social, relational, and structural influences shaping grief. Experiences of racism emerged as a fundamental and cross-cutting influence, affecting interactions with healthcare, support services, and broader social systems. Participants described how cultural norms, migration experiences, faith, community expectations, and access to informal and formal support shaped their bereavement. These findings have informed the development of a new theoretical model, the ‘Culturally Informed Ecological Model of Grief’. To our knowledge, this is the first to demonstrate that grief is a racialised socio-relational experience.

### What this study adds

Participants’ accounts revealed that experiences of racism were inseparable from their bereavement, causing greater, cumulative trauma. Racism experienced in healthcare, workplaces, and even counselling settings compounded the emotional impact of loss, leaving individuals to mourn not only the death of an important person but also the ongoing experience of racial injustice. This finding aligns with theories of intersectionality [31] and disenfranchised grief [32], which highlight how structural inequalities shape the legitimacy and expression of grief. For many, racism eroded trust in institutions, constrained help-seeking, and deepened feelings of alienation. Similar to concepts of cultural and racial trauma [33], participants’ narratives show that bereavement cannot be separated from the social and historical contexts in which it occurs. Recognising this layered form of grief underscores the need for bereavement support services that move beyond ‘cultural sensitivity’ toward ‘cultural safety’ and an explicit acknowledgement of racism as a determinant of grief experience and support access.

Participants frequently used metaphors and analogies to communicate and interpret their grief. These metaphorical expressions reflect processes of sense-making, through which individuals attempt to understand and communicate the impact of loss. This aligns with the ‘Communicated Sense-Making’ model [34], which emphasises the role of narrative and interpersonal communication in constructing meaning following bereavement. Other existing grief models, such as the ‘Dual Process Model’, provide important insights into the psychological processes involved in adapting to bereavement, including emotional distress and coping. Many of these processes were evident in our participants’ experiences and likely reflect common human responses to loss across populations. However, our findings highlight additional structural, relational, and cultural dimensions that are not explicitly addressed. Participants described how experiences of racism, discrimination, migration, and structural inequities influenced their grief experiences, shaping their sense of belonging, trust in institutions, cultural continuity, and access to support. These influences reflect broader forms of loss beyond the death itself, including loss of cultural connection, social recognition, and psychological safety.

The ‘Culturally Informed Ecological Model of Grief’ therefore extends current understandings of grief beyond existing, individualised frameworks. The model incorporates social and structural determinants, enabling grief to be understood as both an individual and socially situated experience. Within the context of bereavement, the five systems of the model – microsystem, mesosystem, exosystem, macrosystem and chronosystem – help visualise the multi-dimensional factors influencing grief and coping mechanisms. At the centre is the bereaved person, recognising the variability in the expression of grief, the interlinked personal and collective community grief and experiences of racism [7]. The microsystem shows the people and resources most immediate to the bereaved person [16]. The mesosystem relates to interactions between the different microsystems in a bereaved person’s life; assumptions can’t be made that these are always positive. This confronts suppositions that specific ethnically diverse communities inherently have better means of informal community support [12]. For those who were experiencing mental health issues, for example or have experienced a stigmatized loss, the relative lack of openness and social judgements could intensify participants’ distress. The exosystem represents formal and informal social structures e.g., wider community networks, existing formal bereavement support systems. Although not always directly interacting with the bereaved person, they may still influence bereavement, usually via the people and resources in the microsystems. The macrosystem incorporates wider society and elements that influence grief e.g., cultural ideologies and attitudes such as gender roles. The chronosystem relates to environmental changes over time. These may include changes in roles, relationships and adaptations to a different life without the bereaved person.

Service providers’ traditional adherence to individualised psychological frameworks may also result in disadvantage and inequity relating to appropriate support. Cognisant with some of our findings, this may mean services do not adequately address culturally specific mourning practices, collective and family-based grieving processes, experiences of racism, or structural barriers such as language, trust, and accessibility. There may be a disproportionate disadvantage arising from unmet religious, spiritual or cultural needs which are specific to certain groups. The absence of collective, community-driven and faith-centred values means such models often fail to recognise ‘*how the continuing aftermath of death is experienced in the everyday*,* relational lives of the living*’ [35]. For example, the African term ‘*ubuntu*’ conveys a view that being human is intricately related to being bound with others – *‘I am because we are one.*’ [36] Hence, a death may be regarded as a threat to the survival of the collective unit [35] or, as seen in our study, represent a loss of cultural knowledge as older generations die. The concept of personal and collective grief being inseparable experiences, due to both historical and ongoing racism, has also been highlighted in the context of the disproportionate experience of loss by Black Americans and UK Black communities [7, 37].

Unlike prior ecological or grief models, the ‘Culturally Informed Ecological Model of Grief’ introduces three novel constructs:

1. structural racism as a cross-systemic determinant of grief – reflecting participants’ pervasive experience of racism through life and into death and bereavement.
2. cultural continuity and collective identity as cross-cutting forces influencing all levels and being both protective yet vulnerable processes – so whilst shared beliefs, traditions and rituals could provide support, this was not universal and could be constraining due to stigmas and expectations.
3. a reflexive loop through which collective responses to loss can transform social systems and enable adaptation and change – for example, one participant sharing how they had set up a support network for others in their community following her own grief experiences.

The ‘Culturally Informed Ecological Model of Grief’ moves beyond traditional paradigms toward approaches that reflect the lived realities of ethnically diverse communities.

### What our findings mean for people and services involved in supporting the bereaved

In many countries, such as the UK, parts of Europe, and North America, ethnic diversity is increasing, and hence, our findings have direct applicability for service provision [38]. Support services need to extend beyond individualised models of counselling. Our study shows how racism pervades through healthcare, employment, social care and wider society, to include the ecosystem of bereavement services. Moving away from cultural competency and instead embedding cultural safety [27] through acknowledging historical injustices, validating collective expressions of grief, and actively confronting racism will be essential in building trust. This is fundamental to influencing perceptions about engagement and access to services [39]. A collective approach to anti-stigma strategies such as public campaigns is needed to help improve health outcomes [40].

**Fig. 3:**
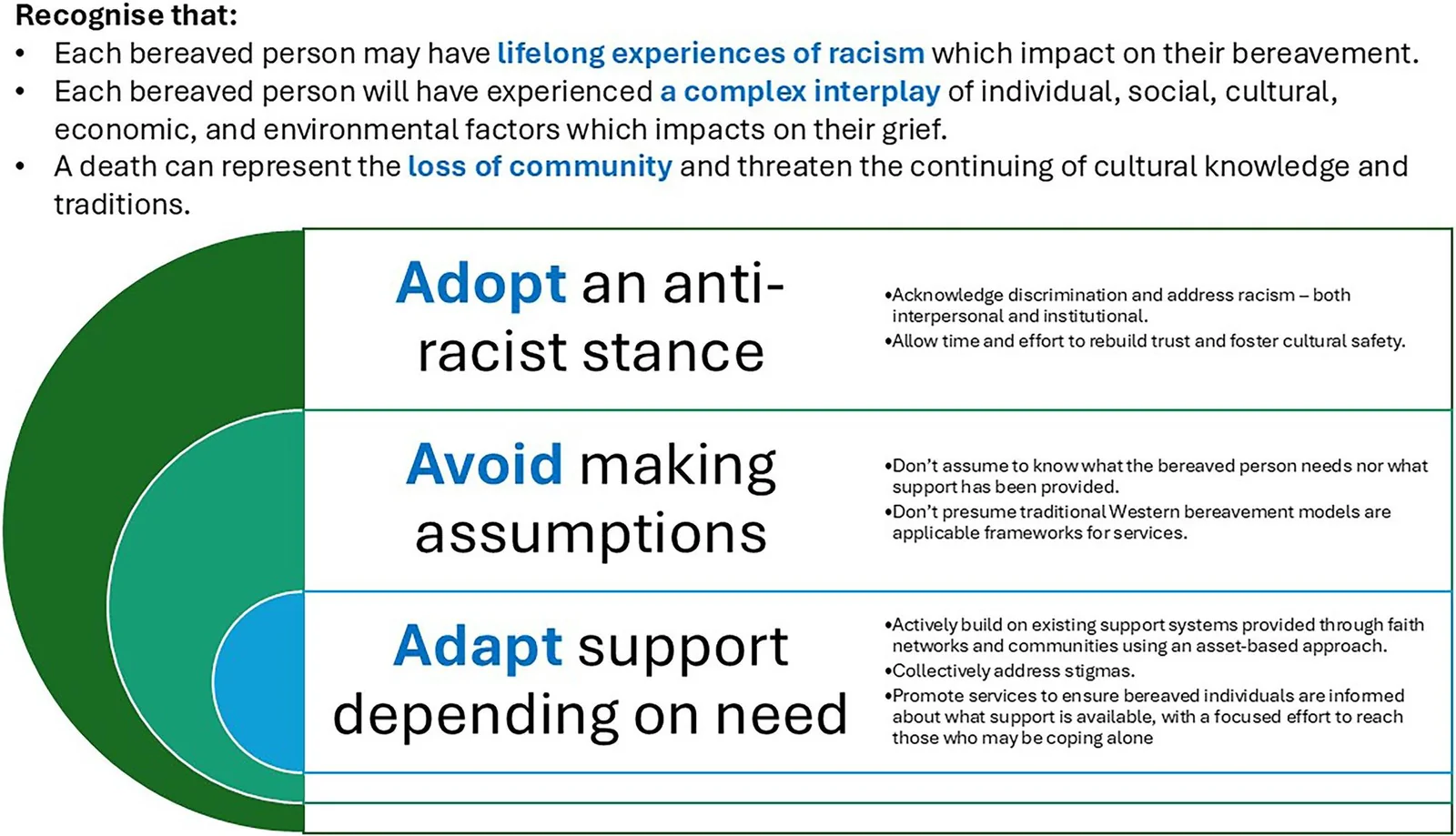
Principles of person-centred care for supporting the bereaved

Bereavement services should universally recognise grief as relational, often embedded in family and community life, while also addressing the structural barriers that limit access to support. This requires flexible models of care that engage with faith leaders, community networks, and culturally specific forms of support alongside formal health services. Additionally, services need to consider individual circumstances and aspects of personal and relational support systems when providing bereavement support. An asset-based approach is recommended to build on the community’s existing strengths and skills [41, 42]. In doing so, bereavement care can better reflect the lived realities of ethnically diverse communities and provide equitable, meaningful support. Our findings inform principles for people and services supporting the bereaved, namely: adopt an anti-racist stance; avoid making assumptions and adapt support depending on need (Fig. 3).

These initial principles will be further developed within the subsequent stages of this research, involving bereavement support services and co-design workshops with key stakeholders, to inform evidence-based, culturally informed recommendations.

### Strengths and weaknesses

To our knowledge, this study is the largest, qualitative study involving ethnically diverse communities in England, and one which has generated a novel theoretical model for grief. The study brings new insights into the understanding of bereavement experiences and confronts previous assumptions. Relative to the UK’s 2021 Census data, our study had good or proportional representation from many different ethnically diverse communities. A striking feature of the cohort was not only its ethnic diversity but also the intersection of multiple dimensions of marginalisation (over two-thirds were first generation migrants, over half had a first language other than English). These groups are often excluded from UK health research, where participation typically requires literacy, English fluency, and familiarity with institutional systems. Our novel approach to recruitment using the ‘Respect-Trust-Equity’ model of recruitment and working in partnership with Community Researchers helped build trust and respect. Their input into data collection, analysis and interpretation of findings undoubtedly enhanced the quality of the study.

There were, however, some limitations. We recognise that our recruitment strategies may have introduced potential selection bias. For example, the use of social media, whilst reaching a much wider geographical area, may have fostered self-selection i.e., people already interested or engaged with bereavement support were more likely to participate. Additionally, recruitment through Community Researchers and community networks may mean those already connected to established community organisations were more likely to participate. Consequently, people who were more socially isolated, less connected to community structures, or who had lost trust in research may have been under-represented in our sample. It is notable that some communities, e.g. Roma, Gypsy or Irish Traveller, Jewish, Arab and mixed ethnic groups, were also under-represented. Additionally, further work is needed to explore those experiencing specific traumatic types of death e.g., murder, manslaughter, as well as the experiences of bereaved children from ethnically diverse communities. Expanding representation from male perspectives would be beneficial and further study of gendered expectations of grief is warranted [43]. It was not feasible to ensure all aspects of intersectionality were considered; hence, our study represents a first step in understanding how grief is experienced and interpreted within certain ethnically diverse communities.

Secondly, we conducted interviews and focus groups in multiple languages, but our reliance on the English language comes with restrictions. Some languages do not have direct terms for ‘grief’ or ‘bereavement,’ [44] which may have affected the communication about our study. Additionally, we recognise that translation involves interpreting culturally embedded realities rather than substituting words verbatim, which can limit or alter meaning making. However, we balanced this risk against our priority to reduce systemic barriers to participation by allowing people to speak in the language of their choice.

Finally, we used both face-to-face and online video means of data collection. Although in-person data collection is generally regarded as the ‘gold standard’, a recent scoping review [45] reported that remotely collected data is likely to generate similar themes to in-person data collection. There is more uncertainty about the impact of data collection format when exploring sensitive topics. Within this study, it was observed that focus groups generally enabled participants to open up and share their experiences. This promoted trust, empathy and understanding from other participants. The exception was within one mixed gender focus group where male participants contributed more than female participants. This suggests gender segregation may be appropriate for some communities. Although interaction between online focus group participants may be reduced, the significance of this for data quality is not known. This contrasts with emerging evidence that participants may be more willing to disclose sensitive information via remote interviews [45]. Whilst balancing practical geographical factors, we aimed to offer as much choice as we could and reduce power hierarchies.

## Conclusions

Our study provides robust evidence that racism is experienced across multiple communities and institutional settings, including healthcare, and that these experiences directly shape and intensify bereavement. By demonstrating that grief cannot be separated from the structural, cultural and historical contexts in which it occurs, the study makes a substantive theoretical contribution. We introduce the ‘Culturally Informed Ecological Model of Grief’, a novel framework that reconceptualises bereavement as a racialised socio-relational experience influenced by structural inequity and collective resources.

The model offers a new lens through which bereavement can be understood, with clear implications for policy and practice. Consistent with the National Health Service (NHS) Race and Health Observatory strategy [46], bereavement support services must move beyond cultural competence toward cultural safety, actively addressing structural racism and rebuilding trust with marginalised communities. The insights generated are nationally significant and have international relevance for societies characterised by growing cultural diversity. The model provides a foundational evidence base to guide the redesign of bereavement support services and to advance more equitable care.

## Supplementary Information

Below is the link to the electronic supplementary material.

Supplementary Material 1: Additional file 1: Topic guides.

Supplementary Material 2: Additional file 2: Summary of focus groups and interviews.

## Data Availability

Anonymised datasets used and/or analysed during the current study are available from the corresponding author on reasonable request.

## Declarations

### Authors’ information

We have included aspects about our positionality within the main manuscript.

## Abbreviations

COREQ: COnsolidated criteria for REporting Qualitative research
FG: Focus Group (number)
KEEPNET: KEech End of life research Partnership NETwork
NHS: National Health Service
P: (interview) Participant
PPI: Patient and Public Involvement
RTE: Respect, Trust and Equity
UK: United Kingdom
USA: United States of America
