# Supplementary material for: Development of the ‘Culturally Informed Ecological Model of Grief’ – a national qualitative study of bereavement experiences among ethnically diverse communities
Source: BMC Med. 2026 Jul 14;24:454. doi: 10.1186/s12916-026-05063-9 (PMC13488016; doi:10.1186/s12916-026-05063-9)
Supplement: Supplementary file 1 [file 12916_2026_5063_MOESM1_ESM.docx]

**Topic Guide for interviews WP1**

**Study Title: Equitable Bereavement Care for All - An inclusive, qualitative study to improve bereavement services for those from ethnically diverse groups**

N.B This guide will be adapted for different types of participants and as data collection progresses, in discussion with the research team and advisors, and to explore perspectives and patterns in the dataset. Not all questions will be relevant to all participant types.

**Administration:** Researcher to thank the participant for taking the time to talk to us, refer to the information previously provided to them, outline what the interview will cover, how long is likely to last and that they will record with their permission, anonymisation, that they don’t need to answer any questions they don’t feel comfortable with, can stop the interview at any point etc. Acknowledge that it’s an emotive area to discuss and if they need to take a break or stop, that’s totally acceptable. If they have a companion with them who has not completed a consent form, seek verbal consent after the recording has started agreeing to the recording and that we use their data as outlined in the information sheet. Also make note of who they are in relation to participant.

In general, do not suggest responses. If a respondent has expressed difficult understanding a question or prompt it may be necessary to offer a brief example to clarify and illustrate. However, this should be done after the respondent has been given ample opportunity to answer spontaneously.

**Example Introduction:**

*Thank you for joining me for this interview. There are four main parts to the interview.*

- *First, I’ll ask if you feel able to share about how you and their family have coped since experiencing their bereavement and ask you to tell me a little bit more about your grief experience.*
- *Then I’ll ask how that event may have affected you and your family and what support you accessed or were provided (if any).*
- *I’ll then ask a little about your views on bereavement support services*
- *Finally I’ll ask you what might help services better meet your needs and the needs of your community.*

*The interview will be about 60 minutes. Please let me know if you find yourself becoming upset as we go through the questions so we can slow down and talk about it. You don’t have to answer any questions you are not comfortable with, and you can take a break at any time. Also, let me know if you have any questions or don’t understand something. Do you have any questions before we start?* *And how would you like me to refer to the person that has died?*

**Interview guide**

1. To better understand the bereavement journey and needs and how this may be influenced by intersectionality (e.g. age, gender, socioeconomic factors)

Example questions:

- ***Do you feel able to tell us about your bereavement? And about the person who was important to you who died?***

***Prompt****: Who was looking after them prior to their death? Do you mind sharing how they died?*

- *What happened after they died? What went well? What could have been better?*
- *How did their death affect you and your family?*
- *How has it been for you since they died?*

***Prompts****: physical, psychological (anxiety, depression/low mood), social (able to work, financial impact, legal challenges), spiritual/religious needs*

- *Do you think your experience is different compared with other members of your family? In what ways?*

***Prompt****: intergenerational differences, gender*

- *What has helped you cope during this time?*
- *Looking back, is there anything that specifically stands out to you about your bereavement? This may be something that went well or was seen as an act of kindness. Alternatively, it could be something that could/should have been better.*

2. Why people within specific communities might not use formal and informal support?

Example questions:

- ***When someone dies, people get help from different places to help them cope. Bereavement is a very personal experience, and may cause many different emotional responses e.g. sad, angry, guilty and sometimes even relief. Do you feel able to share what helped to manage your feelings during this time? If so, where from, and how did you access this help or support? (e.g family, friends, wider community, community group)***
- *What have been the main ways in which you have been helped by this support?*
- *Are there any aspects of this support that have been difficult or could have been better?*
- *Are there any types of support that you would have liked to have and could not get?*
- *Did you get any help from the GP or any organisation?*
- *What was stopping you from accessing support from XYZ?*
- *What made you feel that you couldn’t access support from XYZ?*

3. Appropriateness, accessibility, acceptability of bereavement services.

Example questions:

- ***Sometimes it helps to talk to people when you are grieving. There are services which are available called bereavement services which are available to do this. They have experts trained to do this. Did you access bereavement services? If yes, please tell us about your experience. If no, why not?***
- *Do you think that bereavement services meet your needs, your family’s needs and your (insert ethnic group here collected from questionnaire data prior to interview) community’s needs?*
- *Do you think that there is anything which helps or makes it harder to access bereavement services?*

*Prompts: Do you think it is acceptable to use bereavement services?*

*Language, diverse staff, cultural and religious congruence*

- *What role do you see for bereavement services?*

4. What could be done to make bereavement services better at meeting needs of ED communities.

Example question:

- ***What could be done to make bereavement services better at meeting needs of your (insert ethnic group here collected from questionnaire data prior to interview) community?***

*Is there anything else you would like to mention to us?*

**Wind down:**

Thank participant for their time.

*We've been talking about some difficult topics, and this might have an impact on you for the rest of the day. So I’d encourage you to take it easy if you can, and perhaps think of something you can do to relax or re-charge.*

**Topic Guide for Focus Group WP1**

**Study Title: Equitable Bereavement Care for All - An inclusive, qualitative study to improve bereavement services for those from ethnically diverse groups**

N.B This guide will be adapted for different types of participants and as data collection progresses, in discussion with the research team and advisors, and to explore perspectives and patterns in the dataset. Not all questions will be relevant to all participant types.

**Administration:** Researcher to thank the participants for taking the time to talk to us, refer to the information previously provided to them, outline what the focus group will cover, how long is likely to last, that will record with their permission, anonymisation, that they don’t need to answer any questions they don’t feel comfortable with, can stop the focus group at any point etc. Acknowledge that it’s an emotive area to discuss and if anyone needs to take a break or stop, that’s totally acceptable. If they have a companion with them who has not completed a consent form, seek verbal consent after the recording has started agreeing to the recording and that we use their data as outlined in the information sheet. Also make note of who they are in relation to participant.

In general, do not suggest responses. If a respondent has expressed difficult understanding, a question or prompt may be necessary to offer a brief example to clarify and illustrate. However, this should be done after everyone has been given ample opportunity to answer spontaneously.

**Example Introduction:**

*Thank you for coming together for this discussion today. There are four main parts to the discussion.*

- *First, I’ll ask if anyone from the group feels able to share about how they and their family have coped since experiencing their bereavement and ask you to tell me a little bit more about your grief experience.*
- *Then I’ll ask how that event may have affected you and your family and what support you accessed or were provided (if any).*
- *I’ll then ask a little about your views on bereavement support services*
- *Finally I’ll ask you what might help services better meet your needs and the needs of your community.*

*The focus group will be about 1 hour. We’ll be recording the interview, but everything will be kept anonymous. Please let me know if you find yourself becoming upset as we go through the questions or if you want to take a break. You don’t have to answer any questions you are not comfortable with. You can take a break or stop at any time.*

*Also, let me know if you have any questions or don’t understand something.*

*Do you have any questions before we start? And how would you like me to refer to the person that has died?*

**Interview guide**

5. To better understand the bereavement journey and needs and how this may be influenced by intersectionality (e.g. age, gender, socioeconomic factors).

Example questions:

- ***We appreciate that bereavement can be very hard. Does anyone feel able to share how they and other members in your family have coped during this difficult time?***
- *How did their death affect you and your family?*
- *How has it been for you since they died?*

*Prompts: physical, psychological (anxiety, depression/low mood), social (able to work, financial impact, legal challenges), spiritual/religious needs*

- *Do you think your experience is different compared with other members of your family? In what ways?*

*Prompt: intergenerational differences, gender*

- *What has helped you cope during this time?*
- *Looking back, is there anything that specifically stands out to you about your bereavement? This may be something that went well or was seen as an act of kindness. Alternatively, it could be something that could/should have been better. Does anyone feel willing to share their experiences?*

6. Why people within specific communities might not use formal and informal support

Example questions:

- ***When someone dies, people get help from different places to help them cope. Bereavement is a very personal experience, and may cause many different emotional responses e.g. sad, angry, guilty and sometimes even relief. Does anyone feel able to share what helped to manage their feelings during this time? If so, where from, and how did you access this help or support? (e.g family, friends, wider community, community group)***
- *What have been the main ways in which you have been helped by this support?*
- *Are there any aspects of this support that have been difficult or could have been better?*
- *Are there any types of support that you would have liked to have and could not get?*

*Prompt: psychological, financial, practical, spiritual/religious needs*

- *Did anyone get any help from the GP or any organisation?*
- *What was stopping you from accessing support from XYZ?*
- *What made you feel that you couldn’t access support from XYZ?*

7. Appropriateness, accessibility, acceptability of bereavement services.

Example questions:

- ***Sometimes it helps to talk to people when you are grieving. Services called bereavement services are available to do this. They have experts trained to talk to people about their bereavement and grief. Did anyone access bereavement services?***
- *If yes, please tell us about your experience. If no, why not?*
  - *What was stopping you from accessing support from bereavement services?*
  - *What made you feel that you couldn’t access support from bereavement services?*
- *Do you think that bereavement services meet your needs and your family’s needs?*
- *Do you think that bereavement services meet your needs, your family’s needs and your (insert ethnic group here collected from questionnaire data prior to interview) community’s needs?*
- *Do you think that there is anything which helps or makes it harder to access bereavement services?*

*Prompts: Do you think it is seen as acceptable to use bereavement services? Language, diverse staff, cultural and religious congruence*

- *What role do you see for bereavement services?*

8. What could be done to make bereavement services better at meeting needs of ED communities.

Example question:

- ***What could be done to make bereavement services better at meeting needs of your (insert ethnic group here collected from questionnaire data prior to interview) community?***

*Is there anything else you would like to mention to us?*

**Wind down activity ideas:**

*We've been talking about some difficult topics, and this might have an impact on you for the rest of the day. To finish, can you share one thing you might do later today to look after yourself, relax or re-charge?*
