# Supplementary material for: Development of the ‘Culturally Informed Ecological Model of Grief’ – a national qualitative study of bereavement experiences among ethnically diverse communities
Source: BMC Med. 2026 Jul 14;24:454. doi: 10.1186/s12916-026-05063-9 (PMC13488016; doi:10.1186/s12916-026-05063-9)
Supplement: Supplementary file 2 [file 12916_2026_5063_MOESM2_ESM.docx]

**Additional file 2. Summary of focus groups and interviews**

| **Focus Group**  **N=9 (67 participants)** | | **Interviews**  **N=53** | |
| --- | --- | --- | --- |
| Face-to-face | N=5 | Face-to-face | N=35 |
| Online video | N=4 | Online video | N=18 |
| Language(s) used to conduct focus group | English (n=3), Bengali (n=1), English & Bengali (n=2), Somali (n=1),  English & Chinese (n=1), Bangla (n=1) | Languages used to conduct interview | English (n=43), Cantonese (n=2), Bangla (n=1), Chinese (n=2), English & Bengali (n=2), Polish (n=3) |
| Composition  (mixed gender unless specified) | 1 x Bangladeshi (all women)  1 x Pakistani  1 x Bangladeshi (all men)  1 x Mixed Asian (Bangladeshi, Indian, Pakistani)  1 x Somali  1 x Mixed Asian (Chinese, Hong Kong)  1 x Black African  1 x Mixed Asian (Chinese, Taiwanese, Hong Kong, ‘Other Asian’)  1 x Black Caribbean |  |  |
